# Impact of COVID-19 on Kidney of Diabetic Patients

**DOI:** 10.3390/medicina58050644

**Published:** 2022-05-06

**Authors:** Fahad Abdulaziz Al-Muhanna, Waleed Ibraham Ali Albakr, Arun Vijay Subbarayalu, Cyril Cyrus, Hend Ahmed Aljenaidi, Lamees Ali Alayoobi, Othman Al-Muhanna

**Affiliations:** 1 Nephrology Division, Department of Internal Medicine, King Fahad Hospital of the University, Imam Abdulrahman Bin Faisal University, Dammam 31441, Saudi Arabia; wialbakr@iau.edu.sa (W.I.A.A.); haaljenaidi@iau.edu.sa (H.A.A.); laalayoobi@iau.edu.sa (L.A.A.); 2 Quality Assurance Department, Deanship of Quality and Academic Accreditation, Imam Abdulrahman Bin Faisal University, Dammam 31441, Saudi Arabia; ausubbarayalu@iau.edu.sa; 3 Department of Biochemistry, College of Medicine, Imam Abdulrahman Bin Faisal University, Dammam 31441, Saudi Arabia; ccyrus@iau.edu.sa; 4 Anaesthesia Department, College of Internal Medicine, King Fahad Hospital of the University, Imam Abdulrahman Bin Faisal University, Dammam 31441, Saudi Arabia; othman.almuhanna@yahoo.com

**Keywords:** chronic kidney disease, coronavirus disease 2019 (COVID-19), diabetic kidney disease, diabetes mellitus, human kidney

## Abstract

Given the current state of COVID-19, it is crucial to reveal its evolving relationship with and effect on different body organ systems and their diseases. The severity and outcome of COVID-19 have a very complex relationship, especially to the vital organs including the kidney, either in their state of health or disease. Additionally, it is well known that diabetes affects the kidney, leading to diabetic nephropathy. The kidney is also affected by different pathological and immunopathological reactions with COVID-19 infection, leading to acute kidney injury. Therefore, this review intended to extract the recent advances, updates, and discoveries about the effects of COVID-19 on diabetic patients and the relationship between COVID-19 invasion and the diabetic kidney and to discuss the current state of knowledge that has not yet been proved or disproved, leading to numerous controversial issues in looking for the effect of COVID-19 associated with diabetes mellitus on the human kidney.

## 1. Introduction

In China, numerous cases of novel coronavirus pneumonia (NCP) have been observed since December 2019 [1]. After that, coronavirus disease 2019 (COVID-19) has quickly transmitted universally, resulting in over 110 million confirmed cases and 2.5 million mortalities as of the 16th of February 2021 [2]. It is striking the world in wave patterns of different COVID-19 variants. Individuals affected with the SARS-CoV-2 virus may experience complications that result in lung infection, intubation, and even demise. The World Health Organization stated that those of advanced age and persons with pre-existing clinical illnesses such as diabetes mellitus (DM), chronic kidney disease (CKD), and heart disease are more prone to develop a severe condition due to their contact with COVID-19. Additionally, they demonstrated a high level of morbidity and death [3]. Therefore, it is valuable to perform an in-depth review to search for any relationship between increased mortality in individuals with DM, diabetic kidney disease (DKD), and COVID-19.

Existing research has mainly assessed the relationship between the presence of any chronic heart conditions and COVID-19 associated death, where subtypes of all heart conditions have been collectively investigated [2]. In contrast, no similar studies which explore kidney disease have been carried out. It was reported that kidneys have protein exudation in the Browman’s capsule around glomeruli, degeneration and desquamation of renal tubules’ epithelial cells, and hyaline casts. Acute kidney injuries occurred in the kidney interstitium, which showed the presence of microthrombi and fibrotic foci [1].

Moreover, DM affects 425 million individuals globally and is predicted to increase by over 600 million by 2045 [4]. DKD is a significant cause of morbidity and mortality in diabetes. The estimates state that DKD occurs in 30–40% of DM cases. Furthermore, CKD is related to most causes of cardiac mortality in diabetic cases. Patients with DM are at a higher risk of contracting infections due to immune dysfunction. Additionally, DM cases with DKD report chronic systemic inflammation, leading to the immunosuppressed state that accounts for infectious complications which collectively control the morbidity and mortality related to these cases [5]. Furthermore, a recent study investigated the presence of heterogeneity in the strength of association between heart disease subtypes and in-hospital death. That study also explained the disease course of COVID-19 in hospitalized cases with and without prior cardiac illness from records beginning at hospital admission to discharge or demise, including the occurrence of heart problems. The data were collected using the CAPACITY-COVID registry and the Lean European Open Survey on SARS-CoV-2 infected patients (LEOSS). It was concluded that substantial heterogeneity exists in the strength of association between heart disease subtypes and in-hospital death. Among the persons with cardiac illness, those with severe cardiac failure are more prone to mortality once admitted with COVID-19 [2]. Likewise, no similar study has been conducted to view the association between DKD and COVID-19. This type of study might provide insight to healthcare professionals and the public about how DKD is linked to COVID-19. Hence, this paper conducted a literature review to reveal the current advances, updates, and discoveries about the impact of COVID-19 on patients who have DM and the relationship between COVID-19 invasion and the diabetic kidney.

## 2. Effect of COVID-19 on Diabetic Patients

DM is linked to a high severity of disease and a high risk of death in COVID-19 confirmed cases. It was observed that DM is related to higher disease severity and worse short-term outcomes. Additional robust personal prophylactic plans are recommended for diabetic people, and extra intensive surveillance and management should be considered if those are confirmed with COVID-19, particularly for elderly persons or those with pre-existing clinical conditions [6].

Approximately 34.2 million of the population have diabetes in the US, in which diagnosed account for 26.9 million and undiagnosed for 7.3 million [7]. In Saudi Arabia, the incidence of DM among adults was 18.3%, as per the International Diabetic Foundation (IDF). Additionally, the IDF ranked Saudi Arabia as the seventh-highest nation for new type 1 diabetes cases annually [8]. Commonly, hypertension and severe obesity are comorbidities in diabetic cases. It is uncertain whether DM alone leads to an enhanced risk of morbidity and mortality connected to COVID-19. Less glycemic control is connected with worse outcomes in diabetic cases [9]. Five mechanisms might raise the capability of COVID-19 to influence diabetic patients; those mechanisms are “lessened T-cell function”, “higher affinity cellular binding and efficient virus entry”, “augmented vulnerability to hyperinflammation and cytokine storm”, “reduced viral clearance”, and “the existence of cardiac disease” [10].

Moreover, a study of 13,268 COVID-19 cases demonstrated that diabetes is significantly related to progression of COVID-19. Confirmed cases of COVID-19 with diabetes showed a high risk of disease severity and related mortality consequences. Considering the rapid increase in research information, future meta-analyses which systematically review the literature are necessary to elucidate the relationship between distinct comorbidities and the risk of COVID-19 progression and death [11].

Persons with diabetes are more prone to contracting the viral infection, as observed during waves of preceding diseases. This does not appear to be the case for COVID-19; however, diabetes is more widespread amongst those with severe COVID-19 [12]. In Wuhan, the data from two hospitals with 1561 COVID-19 confirmed cases presented that those with diabetes (9.8%) were more likely to be admitted to an intensive care unit (ICU) or decease [13]. Similarly, a study with a British cohort of 5693 COVID-19 confirmed cases in hospitals stated that the possibility of mortality was more common in those with uncontrolled diabetes [14]; however, such poorer prognoses may result either from diabetes or associated illnesses, and the risk factors have yet to be entirely clarified [12].

Many with COVID-19 do not experience cytokine storms and their symptoms. Some individuals may be more likely to receive cytokine storms from COVID-19 when they have the specific genes that elicit the immune system to respond in this way; currently, this is not definitively known. The severity of a COVID-19 infection is significantly determined by the existence of underlying health conditions [15].

Diabetes leads to a rise in the risk of thromboembolic events, as it is linked to a prothrombotic state. Such a prothrombotic state results from a difference in clotting factors and fibrinolysis. Furthermore, COVID-19 raises coagulation activity. Intravessel coagulation through infection results from endothelial dysfunction related to hypoxia. Anticoagulation therapy in COVID-19 confirmed cases seems to improve disease prognosis [12]. Varikasuvu et al. stated that diabetic COVID-19 cases are more vulnerable to coagulation dysfunction and inflammation when compared to their counterparts. Sensitivity analysis showed the strength of the overall outcome. The involved research harmonized diabetic and non-diabetic sets for overall comorbidities and comorbidities except for CVD and hypertension. However, the outcomes should be understood with the caveat that diabetes may co-occur with other illnesses in COVID-19 cases. Hence, controlled studies are required to isolate the role of diabetes in COVID-19 [11].

Persons with type 1 and type 2 diabetes treated with insulin, or type 2 diabetes not treated at home with insulin who have raised blood sugar in the hospital should have scheduled insulin while they are hospitalized to accommodate the need for high insulin. Before the COVID-19 outbreak, most patients in the emergency care setting were administered IV insulin infusions. However, several institutes have revised and implemented strategies such as scheduled insulin doses and the use of premixed insulin to treat severely ill cases because of staffing anxieties. It is essential to confirm that the patient is getting regular blood glucose monitoring during insulin therapy. COVID-19 cases have variable degrees of insulin requirements during their admission as a result of factors such as, for instance, concomitant drugs (i.e., vasopressors, hydroxychloroquine, steroids) and altering pathophysiologic conditions (namely, acute or chronic kidney injury) [16].

Diabetes leads to a pro-inflammatory homeostatic immune reaction biased towards T helper cell 1 (Th1) and T17 cells and a decrease in regulatory T cells (Treg). Immune dysfunction of diabetes alone or resulting in infection has been observed for various immune cells, including monocytes, macrophages, and CD4^+^ T cells. It was observed that the count of total T cells and CD4^+^ and CD8^+^ T cell subgroups were considerably decreased and functionally exhausted in confirmed COVID-19 cases, particularly among elderly and seriously ill cases requiring ICU admission. Kulcsar et al. [17] presented that diabetic mice showed a prolonged phase of severe disease and late recovery following MERS-CoV infection. This state was credited to a dysregulated immune reaction with reduced inflammatory macrophages/monocytes and CD4^+^ T cells. Therefore, ideal diabetes and intensive glycemic control treatment may aid in stopping the incidence of severe infections and problems associated with DM. It also fights against the enhanced vulnerability of infections resulting from weakened cellular and humoral immunity [6].

Thus, it appears instinctive to guess that this pathogenic disorder could describe the rising drift of cases, hospitalization, and death for individuals with type 2 diabetes during COVID-19 infection. Additionally, lung infection in type 2 diabetes patients seem to be associated with both interleukin (IL)-6 pathways and hyperglycemia. Notably, supporting these concepts, a current and capable experimental intervention using a monoclonal antibody against the IL-6 receptor in Italy appears helpful in severe lung illness and prognosis in COVID-19 confirmed cases [18].

## 3. Association between COVID-19 and Diabetic Kidney

DM and CKD are common illnesses that display a synergistic relationship with early death. CKD has an important global occurrence affecting 7.2% of the universal adult population, with the count intensely raised in older adults [3]. It was reported that type 1 and type 2 diabetic cases who are administered with angiotensin-converting enzyme (ACE) inhibitors and angiotensin II type I receptor blockers (ARBs) demonstrated a considerably enhanced appearance of ACE2. In CKD cases, this enhanced expression of ACE2 facilitates SARS-CoV-2 infection, and management with ACE2 inhibitors might raise the risk of death in emerging severe COVID-19 [1,19,20].

It is well recognized that SARS-CoV-2 targets respiratory cells; however, other organs, namely the heart, ileum, and kidneys, might be damaged by the invaded virus because they have ACE2. It is acknowledged that the kidneys are more prone to damage, consistent with ACE2 expression. Additionally, SARS-CoV-2 is likely to damage the arterial smooth muscle and myocardial cells. As ACE and ACE2 are dissimilar enzymes with two diverse active sites, angiotensin-converting enzyme inhibitors (ACEi) do not impede ACE2. Furthermore, the evidence is unreliable and varies among the different ARBs, through ARBs can motivate ACE2 in experimental models [5]. The regulation and related cellular machinery of ACE2 and related SARS-CoV-2 coreceptors in proximal tubular epithelial cells (PTECs) is highly relevant to kidney health and metabolic and viral illness [21]. Dr. Gilbert’s team reported that the mean ACE2 mRNA level was increased approximately two-fold in the diabetic kidneys compared to healthy controls. The team observed no significant difference in transcript abundance between individuals on medications that block the renin–angiotensin–aldosterone system and those not receiving the medications [22].

Though CKD is not listed as a causative factor for severe COVID-19, it has arisen as the most predominant comorbidity indicating a high risk for severe COVID-19. Moreover, CKD accounts considerably for the severity of COVID-19. This condition currently leads to intensive efforts to enhance the results for the universal CKD cases (850 million) [23].

Furthermore, COVID-19 in diabetic patients is related to an unduly poorer prognosis. Diabetic ketoacidosis (DKA) is a severe problem resulting from diabetes that is characterized by a mortality rate of about 0.67%. A recent study has revealed the natural history of DKA in the existence of coronavirus disease. It explored the impact of COVID-19 on symptoms, clinical progress, and results in DKA patients. COVID-19 seems to impact the usual history of DKA conversely in type 1 and type 2 diabetes. COVID-19 in type 1 diabetes cases demonstrated a high level of hyperglycemia. Type 2 diabetes cases affected with COVID-19 and DKA showed a remarkably high necessity of ICU and high mortality rates. There is a prerequisite for a joint multi-center study to offer further conclusive outcomes [24].

A study by Leon-Abarca et al. observed that DM exerts a more substantial effect on the rates of infection, intubation, ICU admission, and case fatality due to COVID-19 than CKD alone. DKD patients with COVID-19 showed a higher level of morbidity and mortality than those with only CKD and COVID-19. This variation might result from the supplementary impacts of chronic inflammation and immune dysfunction. DKD patients showed highly significant rates of infection with SARS-CoV-2, ICU admission, and case-mortality when compared to patients with CKD alone. The rates of lung infection and intubation were twice that of CKD alone in DKD cases [3]. Another study by Mohamed et al. concluded that CKD was a crucial independent forecaster of COVID-19 death, along with male gender, old age, and high blood pressure. Further studies would examine the effect of COVID-19 on long-term renal function [25].

Diabetes treatment in COVID-19 confirmed cases poses a tremendous medical endeavor, requiring a highly cohesive team method since it is crucial to decrease the risk of clinical problems and demise. Cautious evaluation of the numerous elements that influence worse prognosis with COVID-19 in diabetic cases might help control the present circumstance and allow the health systems to prepare to meet the upcoming encounters efficiently [12]. The situation needs a careful and critical approach if the diabetic patient is invaded with COVID-19 and is also a victim of DKD. A study by Husain et al. stated that some outpatients (32%) need hospital admission; therefore, outpatient monitoring of the high-risk patients in a proper and safe manner would be required and scheduled for by kidney specialists and the health care system [26].

Concerning kidney failure and kidney replacement therapy, dynamic actions should be taken to reveal the risk factors for kidney function damage in seriously ill cases (i.e., poor perfusion and medications). While treating cases with renal failure, the attention should be on the balance of acids, bases, electrolytes, body fluid, and diet; counting nitrogen balance; and the addition of trace rudiments and energies. Continuous renal replacement therapy (CRRT) can be done in seriously ill cases. CRRT is advised for the circumstances such as acidosis, pulmonary edema, or water overload; hyperkalemia; and fluid control in multiple organ dysfunction [1].

Additionally, a recent study in Saudi Arabia observed the clinical features and results of hospitalized COVID-19 cases with or without DM. It observed the occurrence of DM is high among hospitalized COVID-19 cases in a university hospital in Riyadh. While DM cases have more mortality rates than their counterparts, other elements such as smoking, aging, use of β-blockers, congestive cardiac failure, lung infiltrates, high creatinine, and a severe lack of vitamin D seem to be more critical forecasters of lethal outcomes. Persons with acute metabolic dysfunctions, such as high blood glucose on admission, are more likely to receive rigorous care [27]. This finding might be due to the increased incidence of DM among the Saudi population. Additionally, Saudi Arabia has the second-highest rate of DM within the Middle East. It holds the seventh-highest rate of DM globally. It is expected that about seven million of the population are experiencing DM, and nearly three million are pre-diabetic [28]. An initial nationwide study in March 2020 concerning 1519 COVID-19 confirmed cases also presented that high blood pressure (8.8%) and DM (7.6%) were the most commonly noted comorbidities in the context of Saudi Arabia [29].

Lastly, the interconnection between DKD and COVID-19 should initiate more investigation to reveal the degree to which the virus’ precise mechanisms can influence the deterioration of glycemic control. In certain cases, the remarkable growth of hyperglycemic hyperosmolar syndrome or DKA and perhaps, emerging new-onset DM might lead to an acute kidney injury [12]. The role of different vaccines and their relationship to the worsening of diabetes are related to acute kidney injury as it creates an immune response with reported cases of coagulation that need to be further studied.

## 4. Conclusions

In diabetic patients, poor glycemic control is related to worse outcomes. Few mechanisms enhanced the ability of COVID-19 to influence patients with diabetics. Normal kidney functions affected by COVID-19 are of different forms, including acute kidney injury. The kidney of a diabetic patient will be more prone to a significant effect of COVID-19 depending on the stage of DKD. DKD cases showed higher rates of infection (SARS-CoV-2), ICU admission, and demise than cases with CKD alone, and the lung infection and intubation rates doubled. It was inferred that CKD is a crucial forecaster of death in confirmed cases of COVID-19. The significance of prioritizing confirmed COVID-19 cases with CKD in hospitalization led to close observation and kind care. Further research is essential to investigate the effect of COVID-19 on continuing renal function, existence, and quality of life.

## References

[1] Wei P.F. (2020). Diagnosis and treatment protocol for novel coronavirus pneumonia (Trial version 7). Chin. Med. J..

[2] Linschoten M., Uijl A., Schut A., Jakob C.E.M., Romao L.R., Bell R.M., McFarlane E., Stecher M., Zondag A.G.M., van Iperen E.P.A. (2021). CAPACITY-COVID collaborative consortium and LEOSS Study Group. Clinical presentation, disease course and outcome of COVID-19 in hospitalized patients with and without pre-existing cardiac disease—A cohort study across sixteen countries. Medrxiv.

[3] Leon-Abarca J.A., Memon R.S., Rehan B., Iftikhar M., Chatterjee A. (2020). The impact of COVID-19 in diabetic kidney disease and chronic kidney disease: A population-based study. Acta Biomed..

[4] Toniolo A., Cassani G., Puggioni A., Rossi A., Colombo A., Onodera T., Ferrannini E. (2019). The diabetes pandemic and associated infections. Rev. Med. Microbiol..

[5] D′Marco L., Puchades M.J., Romero-Parra M., Gorriz J.L. (2020). Diabetic kidney disease and COVID-19: The crash of two pandemics. Front. Med..

[6] Zhang Y., Cui Y., Shen M., Zhang J., Liu B., Dai M., Chen L., Han D., Fan Y., Zeng Y. (2020). Association of diabetes mellitus with disease severity and prognosis in COVID-19: A retrospective cohort study. Diabetes Res. Clin. Pract..

[7] Center for Disease Control and Prevention (2020). National Diabetes Statistics Report.

[8] Saudi Gazette (2020). Diabetes in Saudi Arabia: Prevalence, Prevention, and Progress. https://saudigazette.com.sa/article/598121.

[9] Rao S., Ali K., Dennis J., Berdine G., Test V., Nugent K. (2020). Analysis of glucose levels in patients hospitalized with COVID-19 during the first phase of this pandemic in West Texas. J. Prim. Care Community Health.

[10] Muniyappa R., Gubbi S. (2020). COVID-19 pandemic, coronaviruses, and diabetes mellitus. Am. J. Physiol.-Endocrinol. Metab..

[11] Varikasuvu S.R., Varshney S., Dutt N. (2021). Markers of coagulation dysfunction and inflammation in diabetic and non-diabetic COVID-19. J. Thromb. Thrombolysis.

[12] Apicella M., Campopiano M.C., Mantuano M., Mazoni L., Coppelli A., Prato S.D. (2020). COVID-19 in people with diabetes: Understanding the reasons for worse outcomes. Lancet Diabetes Endocrinol..

[13] Shi Q., Zhang X., Jiang F. (2020). Clinical characteristics and risk factors for mortality of COVID-19 patients with diabetes in Wuhan, China: A two-center, retrospective study. Diabetes Care.

[14] Williamson E.J., Walker A.J., Bhaskaran K., Bacon S., Bates C., Morton C.E., Curtis H.J., Mehrkar A., Evans D., Inglesby P. (2020). Factors associated with COVID-19-related death using OpenSAFELY. Nature.

[15] Hickman R.J. (2020). What Is Cytokine Storm Syndrome: An Exaggerated and Dangerous Immune Response.

[16] Kimberly E., Rickard J.P. (2020). The effect of COVID-19 on patients with diabetes. US Pharm.

[17] Kulcsar K.A., Coleman C.M., Beck S.E., Frieman M.B. (2019). Comorbid diabetes results in immune dysregulation and enhanced disease severity following MERS-CoV infection. JCI Insight.

[18] Sardu C., Gargiulo G., Esposito G., Paolisso G., Marfella R. (2020). Impact of diabetes mellitus on clinical outcomes in patients affected by COVID-19. Cardiovasc. Diabetol..

[19] Sidorenkov G., Navis G. (2014). Safety of ACE inhibitor therapies in patients with chronic kidney disease. Expert Opin. Drug Saf..

[20] Wan Y., Shang J., Graham R., Baric R.S., Li F. (2020). Receptor recognition by the novel coronavirus from Wuhan: An analysis based on decade-long structural studies of SARS coronavirus. J. Virol..

[21] Menon R., Otto E.A., Sealfon R., Nair V., Wong A.K., Theesfeld C.L., Chen X., Wang Y., Boppana A.S., Luo J. (2020). SARS-CoV-2 receptor networks in diabetic and COVID-19 associated kidney disease. Kidney Int..

[22] Gilbert R.E., Caldwell L., Misra P.S., Chan K., Burns K.D., Wrana J.L., Yuen D.A. (2021). Overexpression of the severe acute respiratory syndrome coronavirus-2 receptor, angiotensin-converting enzyme 2, in diabetic kidney disease: Implications for kidney injury in novel coronavirus disease 2019. Can. J. Diabetes.

[23] Ortiz A., Cozzolino M., Fliser D., Fouque D., Goumenos D., Massy Z.A. (2021). Chronic kidney disease is a key risk factor for severe COVID-19: A call to action by the ERA-EDTA. Nephrol. Dial. Transplant..

[24] Kempegowda P., Melson E., Johnson A., Wallett L., Thomas L., Zhou D., Holmes C., Juszczak A., Karamat M.A., Ghosh S. (2021). Effect of COVID-19 on the clinical course of diabetic ketoacidosis (DKA) in people with type 1 and type 2 diabetes. Endocr. Connect..

[25] Mohamed N.E., Benn E., Astha V., Okhawere K.E., Kom T.G., Nkemdirim W., Rambhia A., Ige O.A., Funchess H., Mihalopoulos M. (2021). Association between chronic kidney disease and COVID-19-related mortality in New York. World J. Urol..

[26] Husain S.A., Dube G., Morris H., Fernandez H., Chang J.H., Paget K., Sritharan S., Patel S., Pawliczak O., Boehler M. (2020). Early outcomes of outpatient management of kidney transplant recipients with coronavirus disease 2019. Clin. J. Am. Soc. Nephrol..

[27] Alguwaihes A.M., Al-Sofiani M.E., Megdad M., Albader S.S., Alsari M.H., Alelayan A., Alzahrani S., Sabico S., Al-Daghri N.M., Jammah A.A. (2020). Diabetes and COVID-19 among hospitalized patients in Saudi Arabia: A single-centre retrospective study. Cardiovasc. Diabetol..

[28] Al Dawish M.A., Robert A.A., Braham R., Al Hayek A.A., Al Saeed A., Ahmed R.A., Sulaiman Al Sabaan F. (2016). Diabetes Mellitus in Saudi Arabia: A Review of the Recent Literature. Curr. Diabetes Rev..

[29] Alsofayan Y.M., Althunayyan S.M., Khan A.A., Hakawi A.M., Assiri A.M. (2020). Clinical characteristics of COVID-19 in Saudi Arabia: A national retrospective study. J. Infect. Public Health.

